# Supplementary material for: Transient infrared nanoscopy resolves the millisecond photoswitching dynamics of single lipid vesicles in water
Source: Nat Commun. 2025 Jul 1;16:6033. doi: 10.1038/s41467-025-61341-9 (PMC12216292; doi:10.1038/s41467-025-61341-9)
Supplement: Supplementary file 1 — Supplementary Information [file 41467_2025_61341_MOESM1_ESM.pdf]

Supplementary information

Transient infrared nanoscopy resolves the millisecond photoswitching dynamics of single lipid vesicles in water

T. Gölz[1], *E. Baù*[1], *J. Zhang*[2], *K. Kaltenecker*[1,3], *D. Trauner*[4], *S. A. Maier*[5,6], *F. Keilmann*[1*], *T. Lohmüller*[2*], *A. Tittl*[1*]

1. Chair in Hybrid Nanosystems, Nano-Institute Munich, Department of Physics, Ludwig-Maximilians-Universität München, 80539 Munich, Germany

2. Chair for Photonics and Optoelectronics, Nano-Institute Munich, Department of Physics, Ludwig-Maximilians-Universität München, 80539 Munich, Germany

3. Attocube Systems AG, 85540 Haar, Germany

4. Department of Chemistry, University of Pennsylvania, Philadelphia, Pennsylvania 19104-6323, United States

5. School of Physics and Astronomy, Monash University, Clayton, Victoria 3800, AUS

6. Department of Physics, Imperial College London, London SW7 2AZ, UK

Email: fritz.keilmann@lmu.de, t.lohmueller@lmu.de, andreas.tittl@physik.uni-muenchen.de

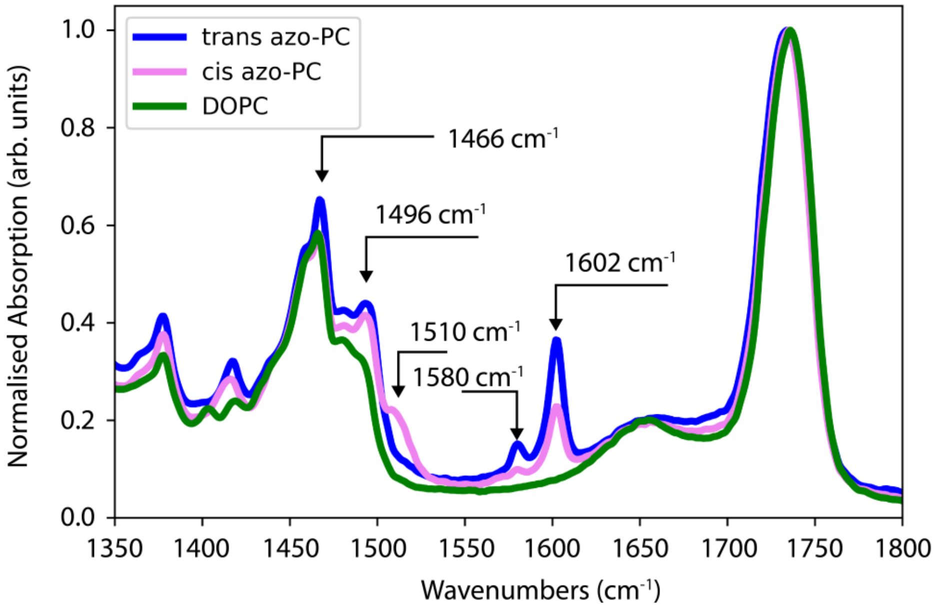

Figure S1: Far-field infrared spectra of the investigated lipid compounds. Normalised ATR-FTIR spectra of pure DOPC (green) and pure azo-PC in the *trans*-(blue) and *cis*-state (violet) measured on dried samples. The data of each spectrum are normalised to the corresponding carbonyl resonance at around 1735 cm^{-1} to compare the peak intensities between the different spectra. The photoswitching between the *cis*/*trans*-state was performed by illuminating the dried sample on the ATR-crystal with 365 nm (*trans* to *cis*) and 465 nm light (*cis* to *trans*).

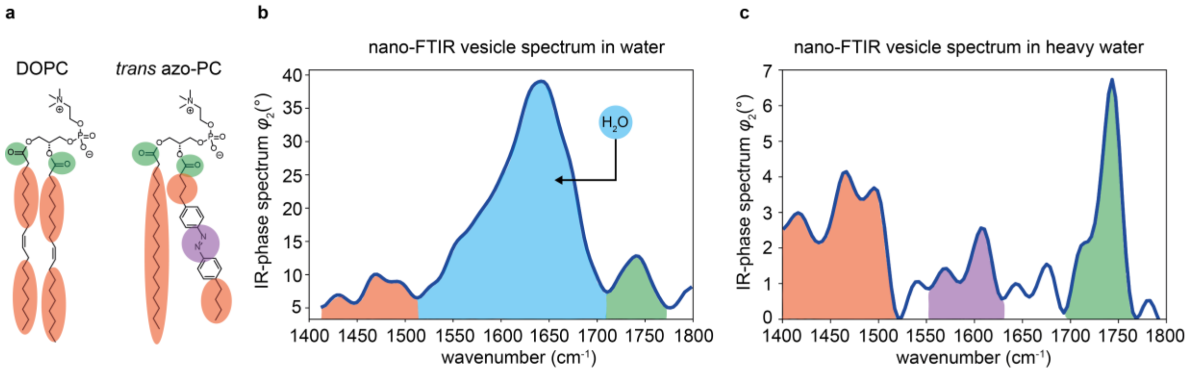

Figure S2: Chemical formula of lipid vesicle compounds and near-field infrared spectra of a vesicle in water and heavy water. (a) Molecular sketches of DOPC and *trans*-azo-PC composing the lipid vesicles with bonds highlighted in specific colours to assign them to the resonances in the nano-FTIR phase spectra (**b, c**). Experimentally determined nano-FTIR phase spectra (φ_2) of a lipid vesicle suspended in H_2O **(b)** and D_2O **(c)**.

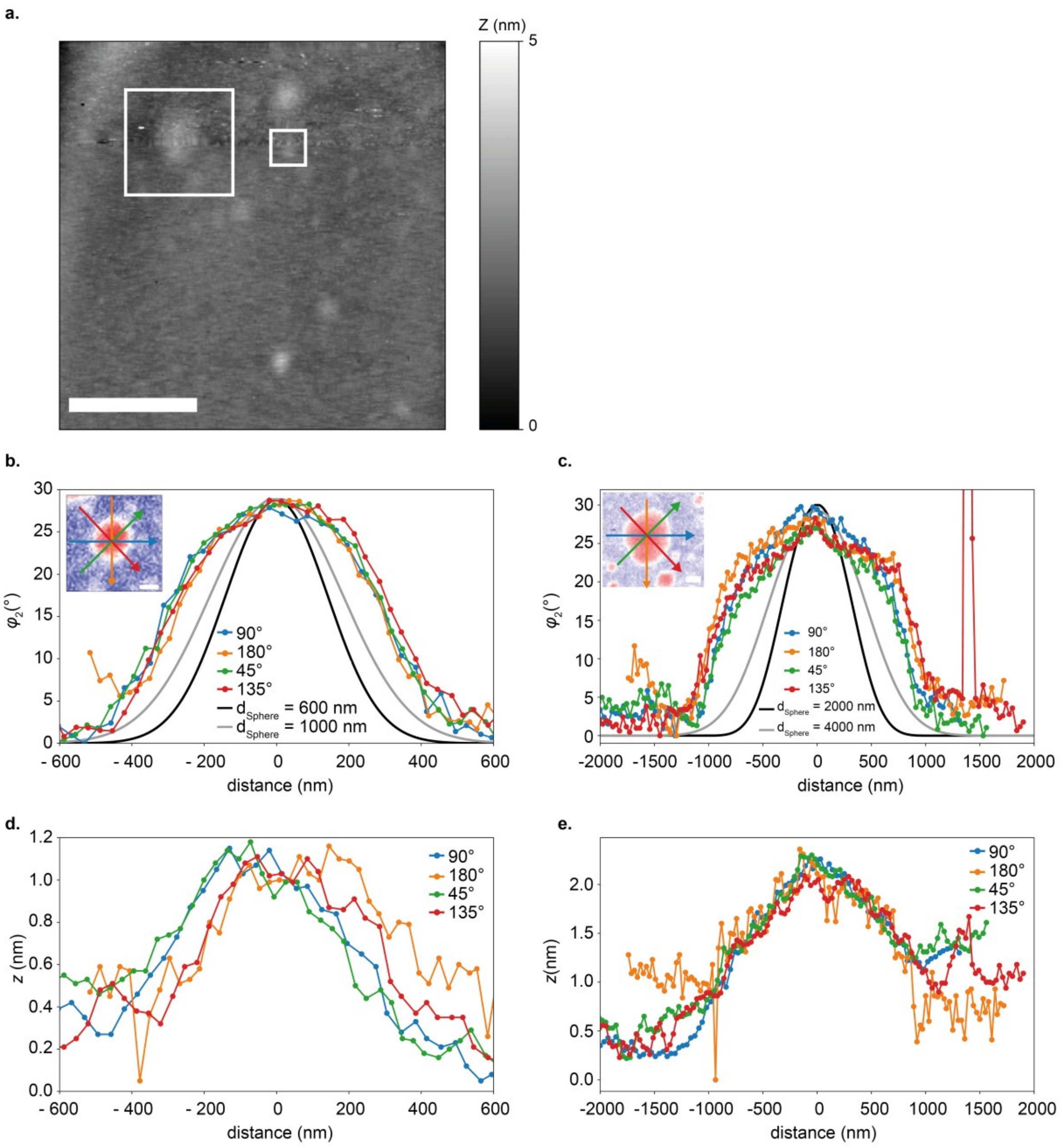

Figure S3: **Topography and near-field phase response of a single lipid vesicle in different directions. (a)** Topographic overview image recorded in corelation to the near-field optical amplitude and phase shown in **Fig. 2a** and **b**. The white boxes highlight the positions of the particles studied with the line cuts in **b – e**, scale bar 5 µm. **(b, c)** Profiles of the green-boxed vesicle and the largest vesicle (next to the brown box) in the phase image shown in **Fig. 2b,** extracted along the arrows indicated in the inset (scale bars 300 nm and 1 µm, respectively). **(d, e)** Correlative topography profiles from the simultaneously measured topographic images (not shown). The theoretical curves in **(b, c)** result from analytically predicting the phase profiles of differently sized, undeformed spheres (d_{Sphere}= 600nm, 1000 nm, 2000 nm and 4000 nm, material PMMA, tip radius = 60 nm and tapping

amplitude a = 80 nm) which are assumed to adhere at one point on the lower surface of a 10 nm SiN membrane (for details see **Methods** and previous literature[1,2]).

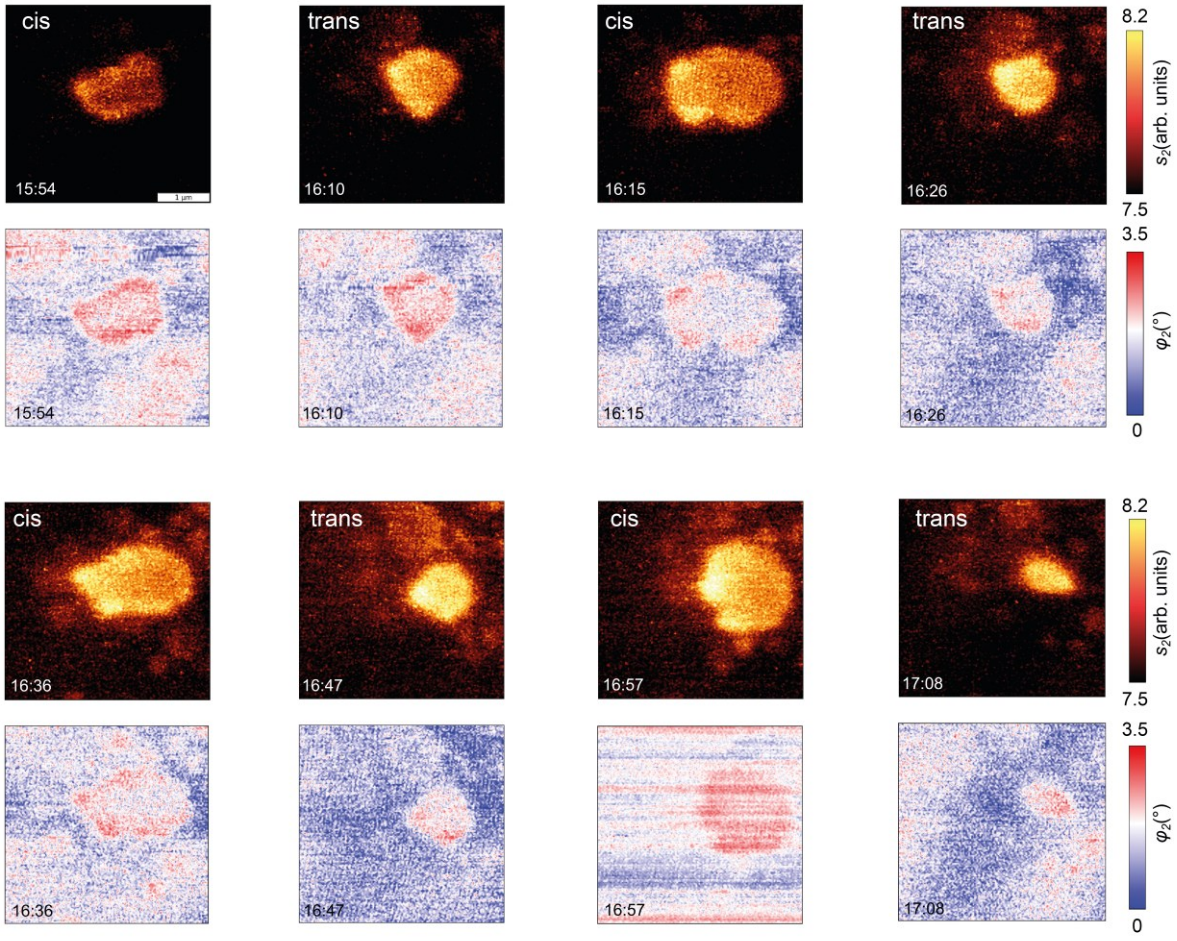

Figure S4: Photoswitching behavior of a single lipid patch. Optical amplitude (s_2) and phase (φ_2) image series of a lipid patch being reversible photoswitched between the *cis* and *trans*-state showing a reversible expansion (*cis*-state) and contraction (*trans*-state) over a 1 h measurement time with a scale bar of 1 µm for all images.

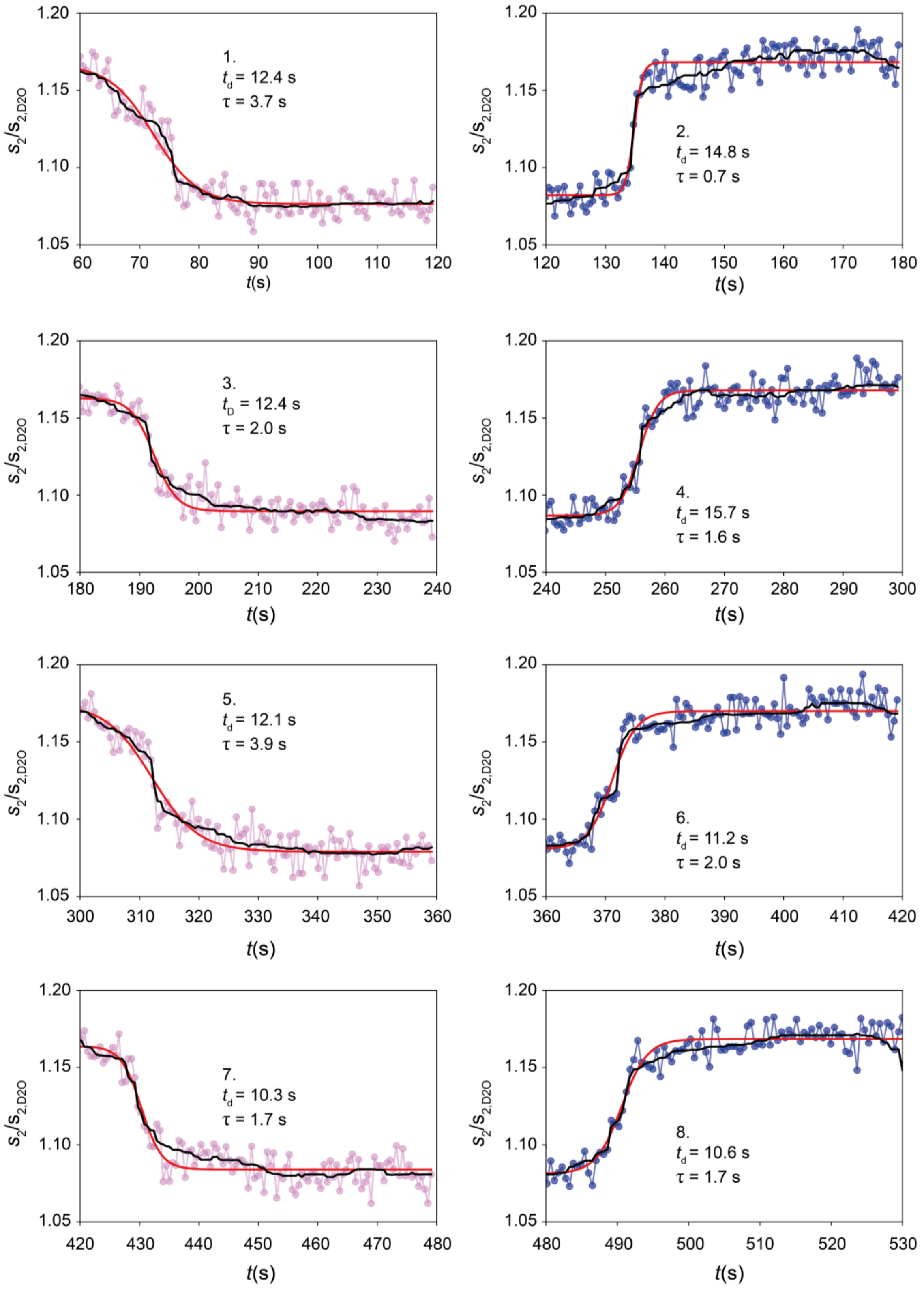

Figure S5: Optical amplitude signal trace of the photoswiching dynamics of a single lipid vesicle. Sigmoidal fit of the transient amplitude signal time trace for investigating the photoswitching dynamic shown in **Fig. 4**. The extracted time constants τ determines the steepness of the switching behaviour describing how fast the system responds. The average τ-value for the *trans*-to-*cis* switching is 2.8±1.1 s, whereas the τ-value for the *cis*-to-*trans* is 1.5±0.5 s, indicating that the *cis*-to-*trans* switching process occurs faster. The delay time t_d, defined as the interval between the switching of the light and the inflection point of the fitted curve, is 11.8±1.0 s for *trans*-to-*cis* switching and 13.0±2.6 s for *cis*-to-*trans* switching. The fitting was performed with a sigmoidal function of the following form $f(t) = \frac{L}{1+e^{-\frac{(t-td)}{\tau}}} + C$.

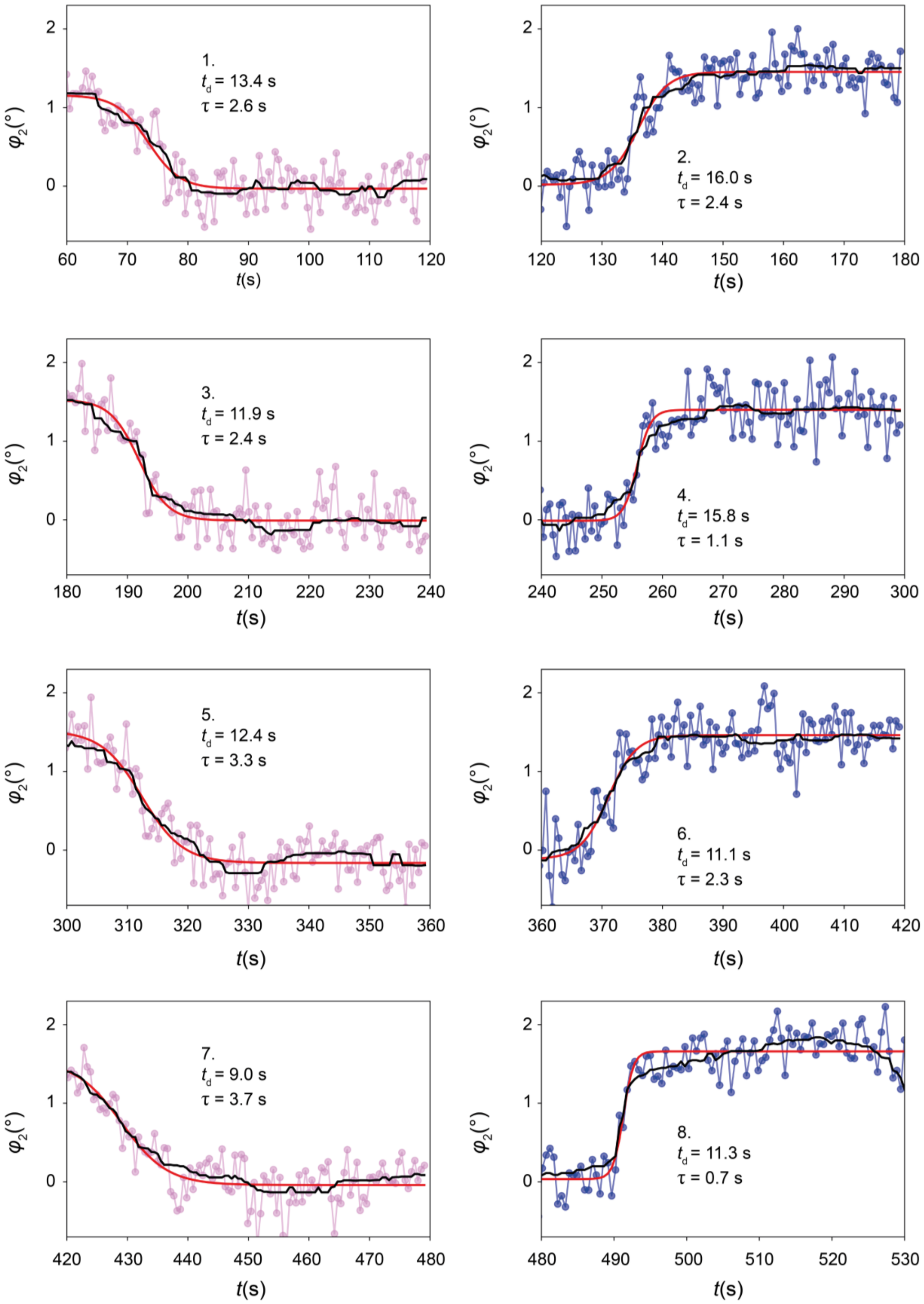

Figure S6: Optical phase signal trace of the photoswiching dynamics of a single lipid vesicle. Sigmoidal fit of the transient phase signal time trace for investigating the photoswitching dynamic shown in **Fig. 4**. The extracted growth parameters τ determines the steepness of the switching behaviour describing how fast the system responds. The average τ-value for the *trans*-to-*cis* switching is 3.0±0.6 s, whereas the τ-value for the *cis*-to-*trans* switching is 1.6±0.8 s, further indicating that the *cis*-to-*trans*-switching occurs faster. The delay time t_d is 11.7±1.9 s for

trans-to-*cis* switching and 13.6±2.7 s for *cis*-to-*trans* switching. The fitting was performed with the same sigmoidal function as in **Figure S5**.

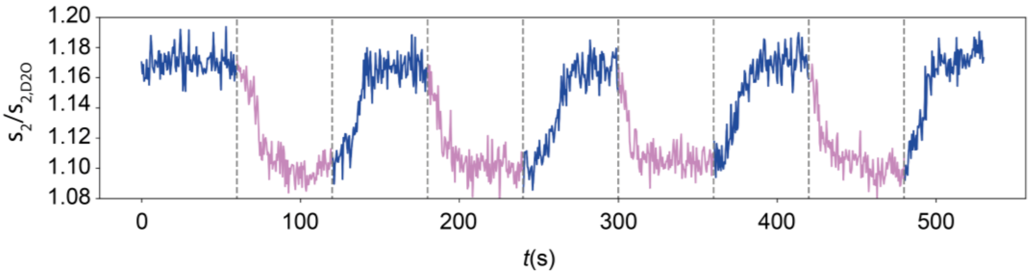

Figure S7: Repeated optical amplitude signal trace on the same lipid vesicle. Near-field time trace showing the normalized optical near-field amplitude ($s_2/s_{2,D2O}$) recorded with 500 ms resolution. The time trace was recorded on the same vesicle as in **Figure 4** reproducing the switching dynamics.

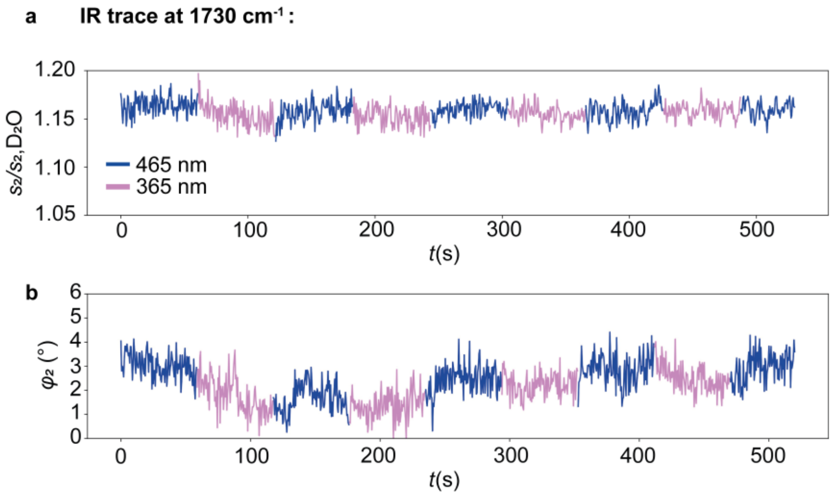

Figure S8: Reference near-field signal trace recorded at the carbonyl resonance on the identical lipid vesicle. Near-field signal trace of the optical amplitude normalised to the D_2O signal ($s_2/s_{2,D2O}$) **(a)** and of the optical phase (φ_2) **(b)** recorded at 1730 cm^{-1} on the vesicle shown in **Figs. 4a, c, d** and **f**. The blue and violet colouring of the IR signal trace specifies the wavelength of the switching light of 465 nm and 365 nm, respectively.

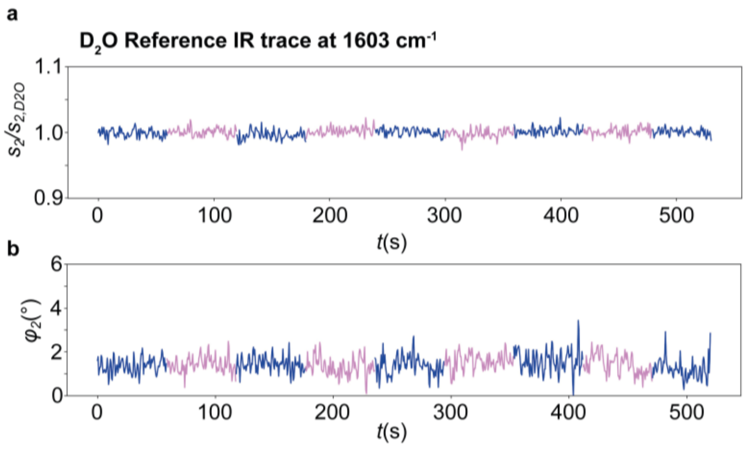

Figure S9: Reference near-field signal trace recorded besides the lipid vesicle to probe the heavy water response. Near-field optical amplitude normalised to the D_2O signal ($s_2/s_{2,D2O}$) **(a)** and phase (φ_2) **(b),** recorded at

1603 cm^{-1} on D_2O next to the vesicle shown in **Figs. 4 a** and **d**. The blue and violet colouring of the trace specifies the wavelength of the switching light of 465 nm and 365 nm, respectively.

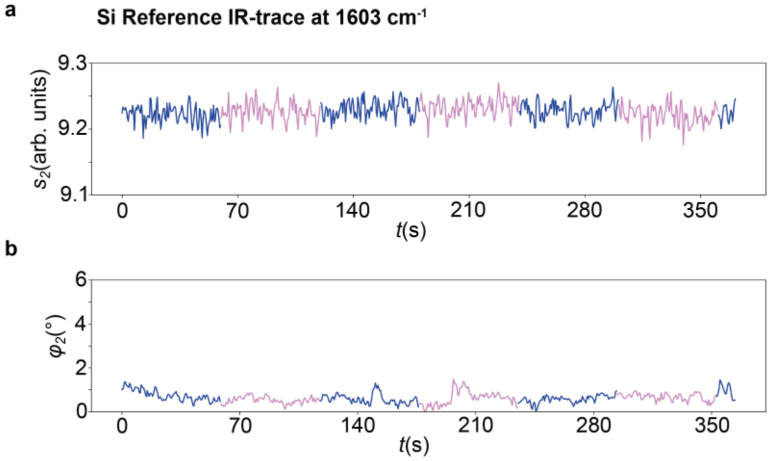

Figure S10: Reference near-field signal trace recorded on silicon surface. Near-field optical amplitude s_2 **(a)** and phase φ_2 **(b)** recorded at 1603 cm^{-1} on a clean Si surface. The blue and violet colouring of the trace specifies the wavelength of the switching light of 465 nm and 365 nm, respectively.

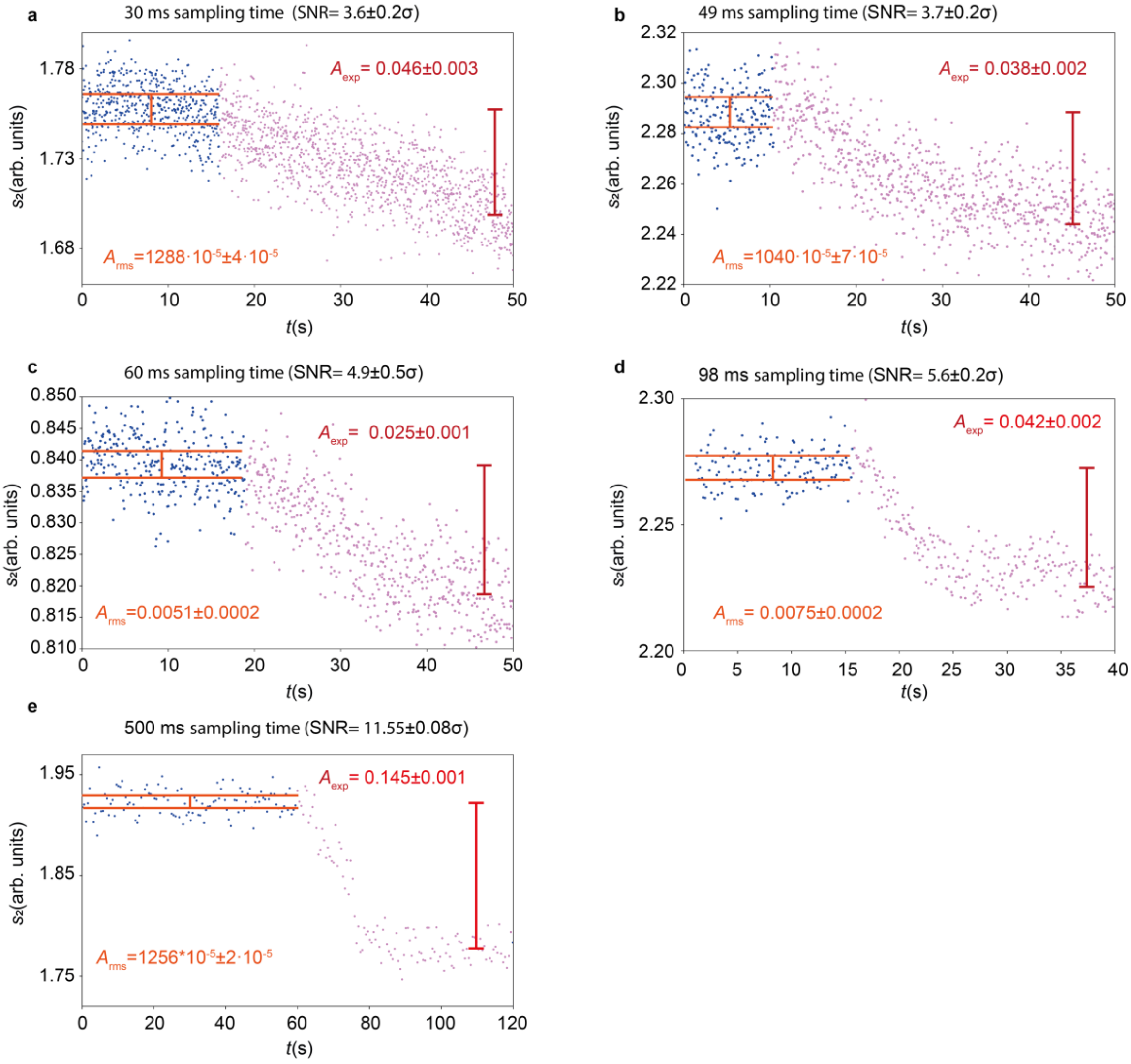

Figure S11: Signal-to-noise metrics of the *trans*-to *cis* switching signal for different sampling times of the near-field signal traces. Near-field time traces of the optical amplitude s_2 recorded with 30 ms **(a)**, 49 ms **(b)**, 60 ms **(c)**, 98 ms **(d)** and 500 ms **(e)** sampling time of the *trans*-to-*cis* switching process. Note that the measurement

at 500 ms was taken on a different vesicle on a different day. The recorded experimental signal values (A_{exp}) are marked by the red bars and the root mean-square of the noise of the steady-state signal (A_{rms}) before the switching perturbation is marked by the orange bars. Based on these values the signal-to-noise values for the photoswitching process have been determined and exceed for all above time-traces the commonly accepted threshold value of 3σ showing that the dynamic photoswitching process can be resolved with 30 ms sampling time.

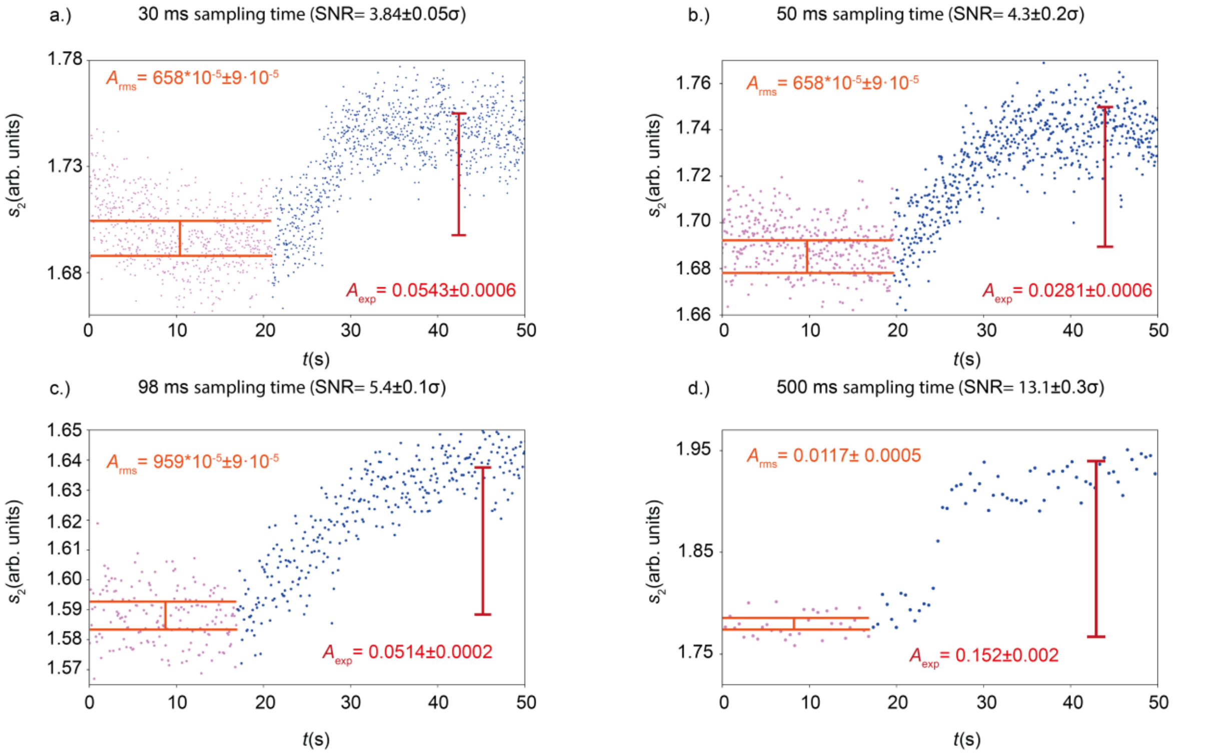

Figure S12: Signal-to-noise metrics of the *cis*-to *trans* switching signal for different sampling times of the near-field signal traces. Near-field time traces of the optical amplitude s_2 recorded with 30 ms **(a)**, 49 ms **(b)**, 98 ms **(c)** and 500 ms **(d)** sampling time of the *cis*-to-*trans* switching process. Note that the measurement at 500 ms was taken on a different vesicle on a different day. The recorded experimental signal values (A_{exp}) are marked by the red bars and the root mean-square of the noise of the steady-state signal (A_{rms}) before the switching perturbation is marked by the orange bars. Based on these values the signal-to-noise values for the photoswitching process have been determined and exceed for all above time-traces the commonly accepted threshold value of 3σ showing that the dynamic photoswitching process can be resolved with 30 ms sampling time.

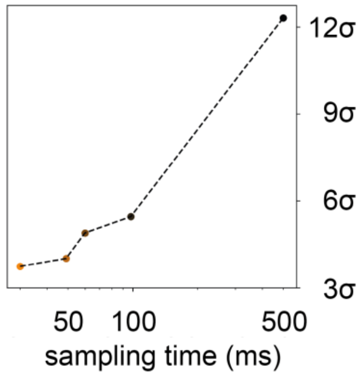

Figure S13: Associated signal-to-noise metrics in relationship to the recorded sampling time of the photoswitching dynamics. The plot relates to the time trace data shown in **Figures S11** and **S12**.

References

1. Baù, E., Gölz, T., Benoit, M., Tittl, A. & Keilmann, F. Nanoscale Mechanical Manipulation of Ultrathin SiN Membranes Enabling Infrared Near-Field Microscopy of Liquid-Immersed samples. *Small (Weinheim an der Bergstrasse, Germany)* **20,** e2402568; 10.1002/smll.202402568 (2024).

2. Kaltenecker, K. J., Gölz, T., Bau, E. & Keilmann, F. Infrared-spectroscopic, dynamic near-field microscopy of living cells and nanoparticles in water. *Scientific reports* **11,** 21860; 10.1038/s41598-021-01425-w (2021).
